# Quantitative Imaging of Gd Nanoparticles in Mice Using Benchtop Cone-Beam X-ray Fluorescence Computed Tomography System

**DOI:** 10.3390/ijms20092315

**Published:** 2019-05-10

**Authors:** Siyuan Zhang, Liang Li, Jiayou Chen, Zhiqiang Chen, Wenli Zhang, Hongbing Lu

**Affiliations:** 1 Department of Engineering Physics, Tsinghua University, Beijing 100084, China, zhangsy92@163.com (S.Z.); jiayou-c16@mails.tsinghua.edu.cn (J.C.); 2 Key Laboratory of Particle and Radiation imaging, Tsinghua University, Ministry of Education, Beijing 100084, China; 3 School of Biomedical Engineering, The Fourth Military Medical University, Xi’an 710000, China, wenlizhang1121@163.com (W.Z.); luhb@fmmu.edu.cn (H.L.)

**Keywords:** X-ray fluorescence, computed tomography, Gd nanoparticles, quantitative image reconstruction

## Abstract

Nanoparticles (NPs) are currently under intensive research for their application in tumor diagnosis and therapy. X-ray fluorescence computed tomography (XFCT) is considered a promising non-invasive imaging technique to obtain the bio-distribution of nanoparticles which include high-Z elements (e.g., gadolinium (Gd) or gold (Au)). In the present work, a set of experiments with quantitative imaging of GdNPs in mice were performed using our benchtop XFCT device. GdNPs solution which consists of 20 mg/mL NaGdF4 was injected into a nude mouse and two tumor-bearing mice. Each mouse was then irradiated by a cone-beam X-ray source produced by a conventional X-ray tube and a linear-array photon counting detector with a single pinhole collimator was placed on one side of the beamline to record the intensity and spatial information of the X-ray fluorescent photons. The maximum likelihood iterative algorithm with scatter correction and attenuation correction method was applied for quantitative reconstruction of the XFCT images. The results show that the distribution of GdNPs in each target slice (containing liver, kidney or tumor) was well reconstructed and the concentration of GdNPs deposited in each organ was quantitatively estimated, which indicates that this benchtop XFCT system provides convenient tools for obtaining accurate concentration distribution of NPs injected into animals and has potential for imaging of nanoparticles in vivo.

## 1. Introduction

Nanoparticle drugs are currently under intensive research for their application in tumor diagnosis and therapy [1,2]. As research on the medicinal property and application of nanoparticle drugs relies on in vivo experiments, a noninvasive detection technique is necessary for researchers to obtain the real-time information of the drugs injected into the animals. Therefore, the approach of nanoparticle detection and quantitative imaging has become a research hotspot in recent years. 

Fluorescence based imaging of nanoparticles is a widely used technique for biomedical imaging [3]. As hemoglobin and water have a lower absorption coefficient in the near-infrared (NIR) region (650–900 nm) compared with the visible region, NIR fluorescence imaging is routinely used for imaging of deeper tissues [4,5,6]. As it is known that characteristic X-rays (also called X-ray fluorescence) stimulated from atoms have much higher energies and penetrability than visible light photons, X-ray fluorescence imaging may further increase the imaging sensitivity of deeper organs.

X-ray fluorescence computed tomography (XFCT), which detects the intensity and position of X-ray fluorescent (XRF) photons emitted from the target element in the object, is considered as a promising approach to obtain the bio-distribution of nanoparticles which include high-Z elements (e.g., iodine (I), barium (Ba), gadolinium (Gd), gold (Au)). XFCT takes advantages of both X-ray imaging and fluorescence imaging. The energies of K-shell characteristic X-rays is sufficiently high (30–70 keV) to penetrate biological tissues, which enables imaging of deep organs. In addition, XRF photons will not be stimulated from background tissues, which significantly enhances the sensitivity of XFCT to the target element [7,8].

XFCT experiments were first performed using synchrotron radiation facilities in order to produce a monochromatic and linearly polarized X-ray source which is ideal for XFCT imaging [9,10,11,12,13]. However, considering the limited access of synchrotron, researchers now focus more on the benchtop XFCT system using conventional X-ray tubes [14,15,16,17,18,19,20,21,22,23,24,25]. Common benchtop XFCT devices use the pencil-beam source to stimulate fluorescent photons and a single-pixel multichannel detector to collect photons emitted from that beam line, then translate the phantom or the pencil beam in steps to acquire the whole projection data of an angle [26,27,28]. This scan strategy measures the projection data with high signal quality and low scattered background but requires a long scanning time. Therefore, a benchtop XFCT system with cone-beam source and detector arrays has been designed to increase the scan efficiency. Simulation studies indicated that the full scanning time can be shortened to less than an hour [14,21,29]. A more practical and cost-effective approach for faster scanning of the XRF signal is to use an array photon counting detector (PCD) instead of multichannel detectors [23,24]. The development of the system design in the past decade demonstrates the potential of XFCT in biomedical research and in vivo imaging [13,30].

Gd-based contrast agents are of interest for their potential in medical applications such as neutron capture therapy (NCT) [31] or magnetic resonance imaging (MRI) [32]. In addition, Gd is also appropriate for benchtop XFCT imaging based on conventional X-ray tubes because the energy of its K-shell characteristic X-rays (42–43 keV) is sufficiently high for biological tissues and its moderate K-edge energy (50.2 keV) enables most of the incident photons to stimulate XRF photons. In the present work, we reported our latest XFCT imaging experiments of a polymethyl methacrylate (PMMA) phantom and three mice using our benchtop XFCT device. Gd nanoparticles (GdNPs) solution which consists of 20 mg/mL NaGdF4 was injected into a healthy mouse and two tumor-bearing mice. The phantom and each mouse were irradiated by a cone-beam X-ray source and a linear-array PCD with pinhole collimation was placed on one side of the rotation stage to record the intensity and spatial information of the fluorescent photons. The results show that the distribution of GdNPs in each target slice (containing liver, kidney or tumor) was well reconstructed and the concentration of GdNPs deposited in each organ was quantitatively estimated, which indicates that this benchtop XFCT system provides convenient tools for obtaining accurate concentration distribution of NPs injected into animals and has potential for imaging of nanoparticles in vivo.

## 2. Results

### 2.1. XFCT Imaging of a PMMA Phantom

**Figure 1:**
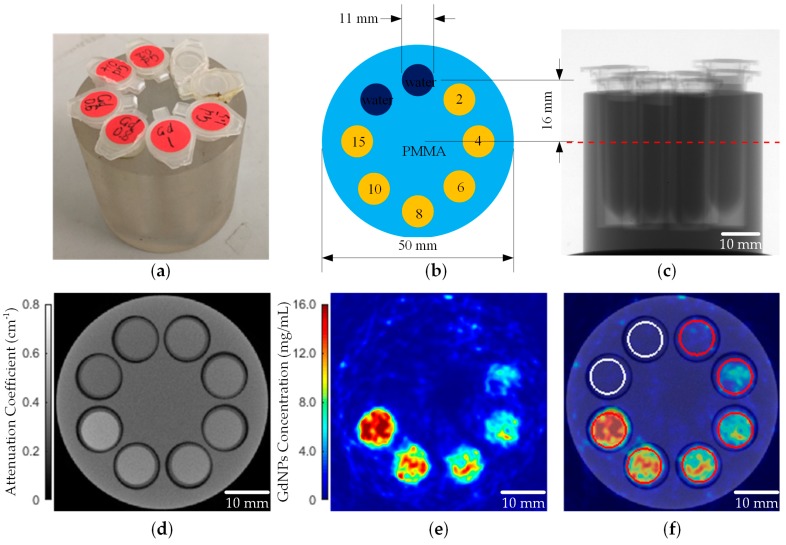
(**a**) The phantom used for concentration calibration; (**b**) The gadolinium (Gd) concentration (mg/mL) of each insertion; (**c**) An X-ray transmission image of the phantom (the red line denotes the axial slice used for the X-ray fluorescence computed tomography (XFCT) scan); (**d**) Reconstructed transmission computed tomography (CT) image; (**e**) Reconstructed XFCT image; (**f**) The CT and XFCT fused image (the red circles denote the sampling points of each insertion and the white circles denote the sampling points of the background).

An XFCT experiment of a phantom with known Gd concentrations was performed to demonstrate the sensitivity and quantitative imaging accuracy of this benchtop system. The phantom (Figure 1a) was made of PMMA with insertions of Gd(NO_3_)_3_ solutions (Figure 1b). The phantom was scanned by Cone-beam computed tomography (CBCT) and one axial slice was selected to perform XFCT scan (Figure 1c). The XFCT image of Gd was reconstructed using maximum likelihood expectation maximization (MLEM) algorithm after scatter correction and attenuation correction (Figure 1e).

**Figure 2:**
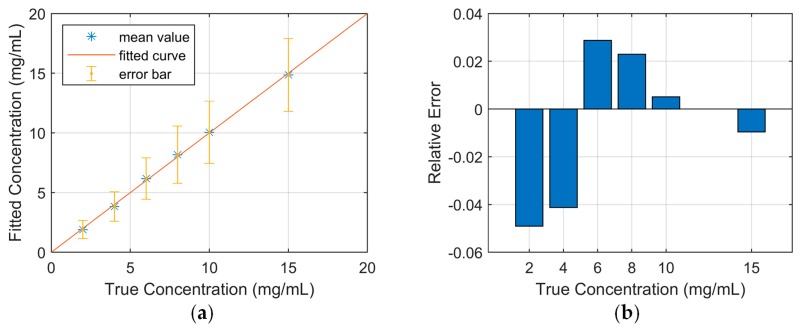
(**a**) Fitted line (*r-square* = 0.9989) and standard deviation about the calibration result; (**b**) Relative error of the fitted result.

The mean pixel values corresponding to each insertion were calculated and the linear relationship between the pixel value and the Gd concentration was fitted by the least square method. The reconstructed results show that the pixel value of the XFCT image is linearly proportional to the concentration of the target element (Figure 2a). As the intensity of the XRF signal depends on the density of the Gd atom, the concentration calibration result of Gd(NO_3_)_3_ in this work is also available for quantitatively determining the concentration distribution of any other drugs containing Gd.

**Figure 3:**
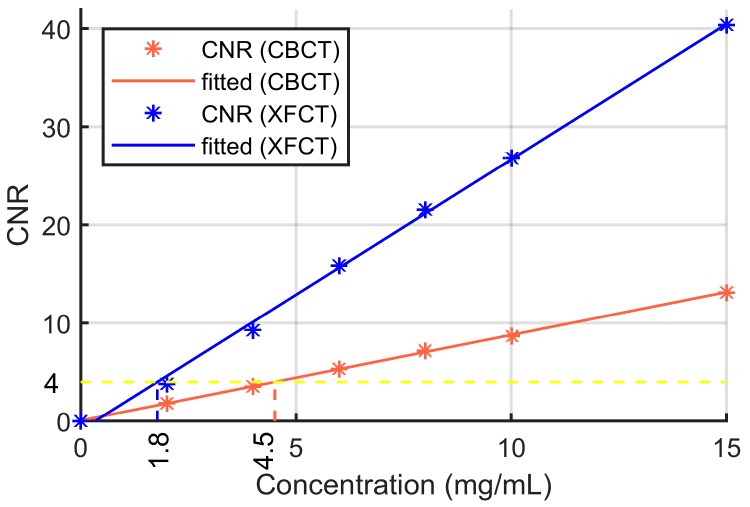
Contrast-to-noise ratio (CNR) of the transmission CT image and the XFCT image.

As XRF photons are only emitted from the target element, background tissues (water and PMMA) will not produce the fluorescent signal, which results in high sensitivity and contrast of XFCT. The contrast to noise ratio (CNR) of transmission CT and XFCT was calculated for quantitative analysis of the image performance (Figure 3). The linear relationship between CNR and the Gd concentration was also fitted by the least square method to estimate the detectability limit of transmission CT and XFCT. According to Rose criterion (CNR > 4) [33], the Gd detectability limit is about 1.8 mg/mL for XFCT and about 4.5 mg/mL for transmission CT in this work.

### 2.2. XFCT Imaging of Mice

A set of XFCT experiments of mice were performed and presented in this work. A healthy mouse and two tumor-bearing mice (with the tumor respectively transplanted in the liver or in the left hind leg) injected with NaGdF_4_ solutions (about 20 mg/mL) were anaesthetized and then fixed on the rotation stage for scan.

**Figure 4:**
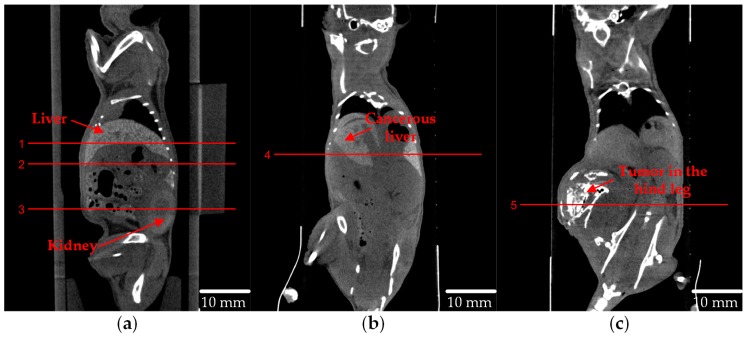
The sagittal plane of (**a**) the healthy mouse, (**b**) the mouse with a tumor transplanted at the liver and (**c**) the mouse with a tumor transplanted on the left leg. The red lines denote the axial slices that was chosen to perform XFCT imaging.

A three-dimension attenuation map of each mouse including 640 axial slices was reconstructed after the cone-beam CT (CBCT) scan. Each slice has 512 × 512 pixels with the size of each voxel being 0.1 mm × 0.1 mm × 0.1 mm. The sagittal planes of each mouse are shown in Figure 4. 

According to the transmission CT results, 5 axial slices, which respectively contained healthy liver, kidneys, cancerous liver or the tumor at the hind leg, were selected to perform the XFCT scan. 

**Figure 5:**
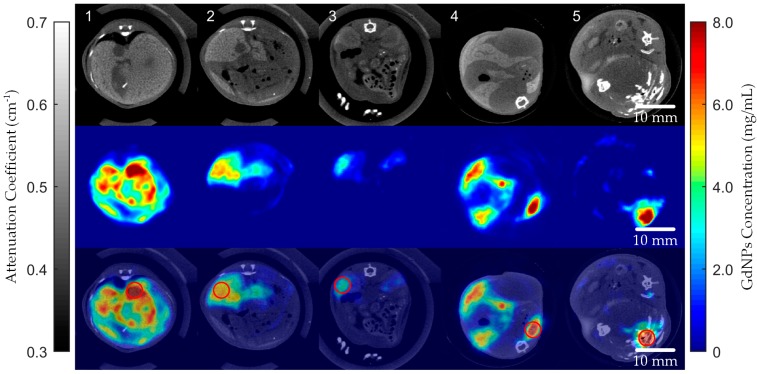
Transmission CT images, XFCT images and CT and XFCT fused images of the 5 target slices. The red circles denote the sampling points of the target regions.

The XFCT images of the five target slices after concentration calibration were obtained and are shown in Figure 5. The quantitative imaging results show that a large amount of GdNPs injected into the mouse were deposited in the liver according to blood circulation. It is noticed that there is also a small part of nanoparticles deposited in the kidneys (about 2 to 3 mg/mL) due to the metabolism. The fusion of transmission CT and XFCT images are also shown in Figure 5 for a more intuitive display of the deposition location.

Different from the phantom imaging, the distribution of Gd deposited in each organ was inhomogeneous. To approximately compare the deposition of GdNPs in different organs, 5 regions with the highest Gd concentrations in each slice were sampled (red circles in Figure 5) and the mean values of the pixels in the sampled regions were calculated (Table 1). Imaging results show that the transmission CT image has higher spatial resolution which provides more detailed structure information, while the sensitivity of the target element (Gd in this work) in the XFCT image is generally higher due to its lower background.

## 3. Discussion

Different from common optical imaging methods which observe objects from a given viewpoint, each pixel in the XFCT images represents the signal intensity of the target element at corresponding voxel in the object. Similar to other emission CT methods (e.g., single-photon emission computed tomography (SPECT), positron emission computed tomography (PET)), the intensity of XRF signal emitted from a certain voxel is proportional to the concentration of the fluorescence probe at this position, which means that the actual concentration of NPs at any position inside the object can be quantitatively obtained by XFCT. It is noticed that the reconstructed values of XFCT images are accurate only when the scatter background is removed and the attenuation of incident beam and XRF photons in the object are corrected. Therefore, the data acquisition procedure and reconstruction algorithm applied for XFCT image reconstruction in this set of experiments were also demonstrated in this work (Section 4.5). The phantom imaging results show the high linear relationship between the concentrations of target element and the pixel values in the reconstructed XFCT image. The XFCT images of 5 target slices in mice were also successfully reconstructed and the concentrations of GdNPs in different organs were quantitatively estimated, which indicates the potential of our XFCT system for high sensitivity and quantitative imaging of NPs in vivo.

Recent development in system design and reconstruction method of benchtop XFCT has increased its sensitivity to NPs, which indicate the potential for quantitative imaging of NP distributions in vivo [30]. One improvement which may further increase the practicability of benchtop XFCT is to shorten the scanning time. Most benchtop XFCT devices in previous studies prefer to use single-pixel multichannel detectors to record the whole spectrum of the secondary X-rays emitted from a beam line in the object. This kind of system acquires a single line integral at a time and obtains the full projection data of an angle by translating the object or the detector. This method is accurate and can discriminate various fluorescent materials at one time but results in long scanning time. The use of pinhole collimation and array detector enables acquiring a projection of the whole object in one scan, which significantly increases the scanning efficiency [21,23]. When acquiring a P (angles) × D (pixels) projection, there would be an approximately D-fold reduction in scanning time when using an XFCT device with a fan-beam source and an array detector. Compared with previous XFCT experiments of Gd using a pencil beam source (i.e., obtaining a 30 × 31 projection data in 4.6 h [28]), the total scanning time in this work was much shorter (7.5 min per slice) and the spatial resolution of the projection data was relatively higher (45 × 256) as a result of the fan-beam scanning strategy. The detection efficiency could be further increased by using a multi-pinhole collimation design [34] when the detection area is sufficiently large.

In this work, the CBCT scan and the XFCT scan were performed separately because the incident beam energy of XFCT is too high (140 kV tube voltage) for CBCT to discriminate tissues in vivo. This problem can be solved by replacing current scintillation flat panel detector with another array PCD to measure attenuation information of the spectrum. Therefore, the CBCT scan and XFCT scan could be performed simultaneously using the same X-ray source, which will result in a reduction of the total scanning time (120s per object in this work). Furthermore, by taking advantage of the pinhole collimation design, current 2D XFCT device can be easily extended to become a 3D device by just replacing current linear array detector with a panel detector when the data acquisition procedure and scanning time remain unchanged. 

Multimodal imaging is considered as one of the promising technologies where the advantages of NPs are maximized [35]. In this work, the transmission CT images with high resolution provided the structural information while the XFCT image enables high contrast quantitative imaging of drug deposition in the organs. Actually, NaGdF4, which was used as X-ray fluorescence probes in this work, is also widely used in optical imaging after doping with ions (e.g., Nd^3+^, Eu^3+^) [36,37]. Therefore, a multimodal imaging method including optical tomography, transmission CT and XFCT might be realized in future: Cone-beam transmission CT provides high resolution structural information of the experimental animal injected with NPs, while optical tomography obtains the in vivo whole body image of NPs and XFCT obtains the quantitative distribution of NPs in the target organs.

One main shortcoming of the current benchtop XFCT device that limits further sensitivity improvement of XFCT images is the bad energy response of recent array PCDs. Due to the charge-sharing effect, a part of scattered photons with higher energies will also be recorded by the target energy bin, which strongly disturb the fluorescence signal. Although the expectation value of these scattered photons recorded by each energy bin can be well estimated by energy calibration and spectrum reconstruction, the statistical noise of these photons will add to the data of the target bin, which reduces the signal-to-noise ratio of fluorescence signal. One solution is to use a PCD with anti-charge-sharing mode. This mode is achieved by coincidence measurement and will reduce the energy measurement error of array detectors caused by charge-sharing effect. Another solution is to use polarized X-ray source or use X-ray polarizers to produce a polarized beam [38]. When the object is irradiated by a polarized beam, the scattering into a detector can be minimized by placement of the detector orthogonal to the polarization direction. Scatter reduction is one of the most important problems in the current XFCT study and will be the focus of our future work.

## 4. Materials and Methods 

### 4.1. Benchtop XFCT Device

**Figure 6:**
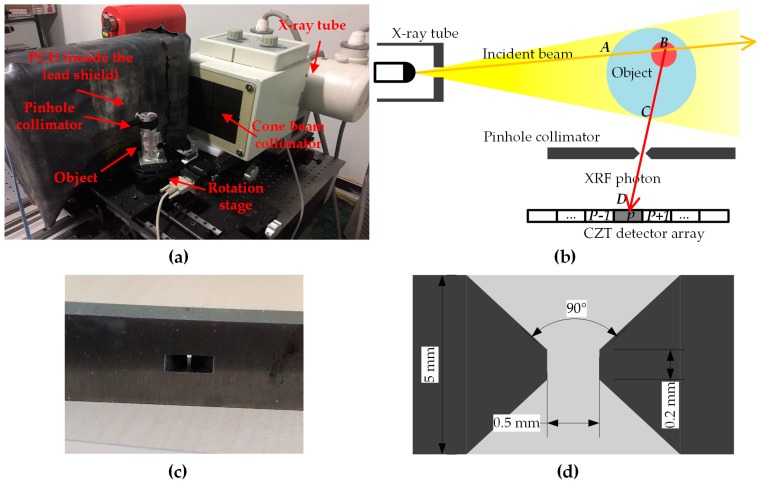
(**a**) The benchtop XFCT device; (**b**) The transport process of the X-ray fluorescence (XRF) signal; (**c**) The front view of the pinhole; (**d**) The cross section of the pinhole.

The XFCT experiments in this work were performed on a benchtop XFCT device which is composed of a conventional X-ray tube, a rotation stage and a CdZnTe linear array detector with a single-pinhole collimator made of tungsten (Figure 6a). The particle transport process of the XFCT system is shown in Figure 6b. The fluorescence material in an arbitrary point *B* will be stimulated by the incident photons which enter the phantom at point *A* and then emit the XRF photons. Some of these XRF photons will finally be collected by the detector array at point *D* after leaving the phantom at point *C* and passing through the pinhole collimator.

### 4.2. Animal Handling and Tumor Generation

Animal manipulations were approved by the Animal Experiment Administration Committee of the Fourth Military Medical University and were performed in accordance with the National Institutes of Health guide for the care and use of laboratory animals. Animal experiments were approved by the Laboratory Animal Research Center of Tsinghua University (Animal Protocol (AP) code: 17-LL1, AP date: 27/09/2017–27/09/2020). Three male nude mice were used for imaging experiments in this study. Each mouse was about 10 weeks old and weighed about 20 g when carrying out the experiments. The first mouse was healthy and the other two mice were transplanted with HepG2 cancer cells (1 × 10^5^ cells/100μL) after birth. The tumor was transplanted in the liver of the second mouse and was transplanted in the left hind leg of the third mouse.

Each mouse was first anaesthetized by intraperitoneal anesthesia of avertin. For the first and second mouse, about 0.3 mL NaGdF_4_ solution was then injected via the tail vein 5 min later. For the third mouse with the tumor in the left leg, the drugs were directly injected into the tumor. Each mouse was then propped upright and fixed on the rotation stage for scan about 5 min later after injection.

### 4.3. NaGdF_4_ NPs

The type of the nanoparticle drug used in the experiment is NaGdF_4_ with surface modification of 2-Aminoethylphosphonic acid (AEP) dissolved in physiological saline. The concentration of Gd is about 20 mg/mL and the diameters of the particles are about 9–11 nm. The NPs were synthesized via a coprecipitation route and the surface NH2-functionalization was carried out by using a modified ligand exchange strategy. The synthesis and surface modification of NaGdF_4_ used in the experiment was similar to those of β-NaGdF_4_: 15% Tb^3+^ NPs presented in the previous work [39].

### 4.4. Cone Beam CT and XFCT

The whole experimental procedure of each mouse included two steps. In the first step, a CBCT scan was performed and a three-dimensional attenuation map was reconstructed. The purpose of the first step was to select several slices which included the target organs and provided attenuation information for quantitative reconstruction of the XFCT images. In the second step, the XFCT scan of each target slice determined in the first step was performed and the XFCT image of each slice was obtained.

**Figure 7:**
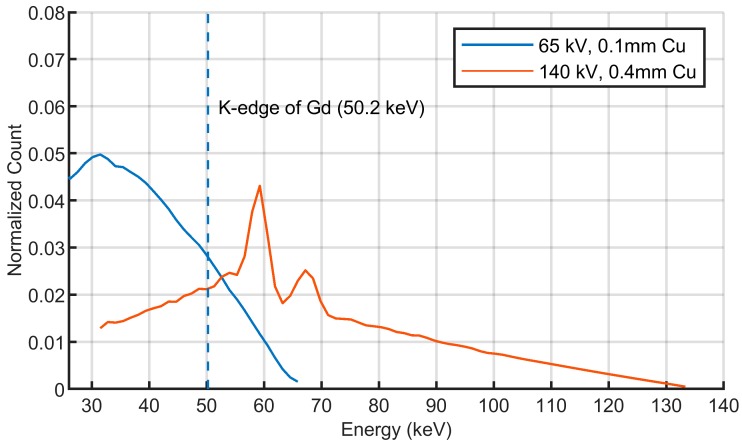
Incident spectrum for Cone-beam computed tomography (CBCT) (65 kV, filtered with 0.1 mm Cu) and XFCT (140 kV, filtered with 0.4 mm Cu) measured by eV-3500 detector.

For the CBCT scan, the incident beam was produced by a low-power micro-focus X-ray tube (65 kV, 0.19 mA, L12161-07, Hamamatsu). The tube voltage was relatively low for better discrimination of different tissues in mice. The incident beam was filtered with 0.1 mm Cu (Figure 7) to reduce artifacts and noises caused by X-ray photons with low energies. The attenuation information was collected by a flat-panel detector (Varian 2520) which had 960 × 768 pixels with the size of each pixel being 0.25 mm × 0.25 mm. Each mouse was rotated 360° at a constant rate of 3°/s and 600 projections were acquired with the exposure time of 0.2 s per projection. The traditional Feldkamp-Davis-Kress (FDK) method [40] was applied for the image reconstruction. The absorbed dose was estimated based on the incident spectrum simulated by SpekCalc [41] and the mass energy-absorption coefficient database from National Institute of Standards and Technology (NIST) [42]. The estimated absorbed dose of CBCT was about 0.027 cGy per slice at the center of the phantom and was about 0.044 cGy per slice at the center of each mouse.

For the XFCT scan, in order to produce the XRF signal with high intensity, each object was irradiated by the incident beam with higher power (140 kV, 25 mA, G-297, Varian) and the beam was filtered with 0.4 mm Cu (Figure 7) to increase the proportion of photons with energies above the K-edge of Gd (50.2 keV). The fluorescent photons were collected by a linear array CdZnTe detector (eV-3500) which had 256 pixels with the size of each pixel being 0.5 mm × 2 mm. The tungsten collimator between the rotation stage and the detector showed a rectangular pinhole 0.5 mm wide and 2 mm high. In this study, the distance between the pinhole and the rotational center was 75 mm, and the distance between the pinhole and the detector was 63.5 mm, which ensured that the diameter of field-of view was larger than 50 mm. For each target slice, 45 projections were obtained for image reconstruction with exposure time of 10s per angle. The estimated total absorbed dose of XFCT was about 45.5 cGy per slice at the center of the phantom and was about 61.0 cGy per slice at the center of each mouse.

### 4.5. Data Acqusition and Image Reconstruction

In this section, we briefly demonstrate the data acquisition and image reconstruction algorithm used in this work. More details about the particle transport process of XRF photons and the scatter correction method have been presented in our previous work [43].

**Figure 8:**
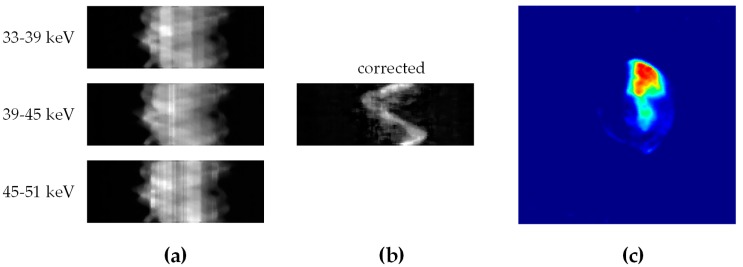
Schematic representation of data acquisition and image reconstruction procedure: (**a**) Raw data recorded by three energy bins; (**b**) projection data after scatter correction and (**c**) the reconstructed XFCT image.

The linear array PCD was operated on the energy-bin mode to collect fluorescent photons passing through the pinhole. The energy bins of the detector were respectively set at 33–39 keV, 39–45 keV and 45–51 keV in this study. The middle energy bin was used to record the fluorescent photons of Gd (Kα1 = 42.98 keV, Kα2 = 42.28 keV) while the other two bins were used for scatter correction (Figure 8a). The number of scattered photons in 39–45 keV energy bin can be estimated as follows:

(1) $$Sca_{39-45keV}=\left(\frac{r_{39-45keV}}{r_{33-39keV}}Ns_{33-39keV}+\frac{r_{39-45keV}}{r_{45-51keV}}Ns_{45-51keV}\right)/2$$

where *r*_33-39*keV*_, *r*_39-45*keV*_ and *r*_45-51*keV*_ are the theoretical relative numbers of scattered photons in each energy bin, *Ns* is the number of photons reaching the detector with energies in the range of each energy bin. 

According to the Klein–Nishina formula, the Compton cross-section is given by

(2) $$f_{KN}\left(E,\theta \right)=\frac{d\sigma }{d\theta }=2\pi r_{0}^{2}sin\theta {\left(\frac{1}{1+\alpha \left(1-cos\theta \right)}\right)}^{2}\left(\frac{1+\cos^{2}\theta }{2}\right)\left(1+\frac{\alpha^{2}{\left(1-cos\theta \right)}^{2}}{\left(1+\cos^{2}\theta \right)\left[1+\alpha \left(1-cos\theta \right)\right]}\right),$$

where *r*_0_ is the classical electron radius and *α* = *E*/(*m*_0_*c*^2^). Assume that the scattered photons reaching the *i*-th pixel have the same scatter angle, the theoretical relative numbers of scattered photons in Equation (1) can be expressed as

(3) $$r_{E\min-E\max}\left(i\right)=\int_{E\min}^{E\max}I_{0}(E)f_{KN}\left(E,\theta_{i}\right)dE$$

where *I*_0_(*E*) is the incident spectrum. Therefore, the fluorescence signal (Figure 8b) can be expressed as

(4) $$N_{XRF}\left(i\right)=Ns_{39-45keV}\left(i\right)-Sca_{39-45keV}\left(i\right)$$

The XFCT image (Figure 8c) was reconstructed using the well-known MLEM algorithm [44]. The formula of each iteration can be expressed as follows:

(5) $$f_{j}^{\left(k+1\right)}=\frac{f_{j}^{\left(k\right)}}{\sum_{i=1}^{n}a_{ij}}\sum_{i=1}^{n}\frac{g_{i}}{\sum_{j^{\prime }=1}^{n}a_{ij^{\prime }}f_{j^{\prime }}^{\left(k\right)}}a_{ij}$$

where *g_i_* is the projection data and *a_ij_* is the projection matrix which is determined by pinhole response function (PRF) of the XFCT device and attenuation of the imaging object. According to the particle transport process shown in Figure 6b, the projection matrix can be expressed as

(6) $$a_{ij}=\Omega_{B\to p}e^{-\int_{A}^{B}\mu \left(E_{inc},l\right)dl}e^{-\int_{B}^{C}\mu \left(E_{xrf},l\right)dl}$$

where Ω*_B→p_* is the PRF of the system which can be easily calculated according to the system geometry and μ(*E*,*l*) is the attenuation map of mice which was estimated by a simple material decomposition method based on transmission CT and XFCT. We assume that the object can be decomposed into three materials: Target NPs (NaGdF4), bone and water. Consider that bone has a much higher linear attenuation coefficient than other soft tissues, the bone region can be segmented according to the effective linear attenuation coefficient. The density distribution of the target element can be determined by the XFCT image without attenuation correction. After material decomposition, the attenuation coefficient of each component at any energy is calculated respectively and then the 2D attenuation map of each target slice is acquired for calculation of the projection matrix.

## Figures and Tables

**Table 1:** Concentration of GdNPs in different organs.

Slice	Organ	Highest Concentration (mg/mL)
1	Noncancerous liver	7.94
2	Noncancerous liver	5.30
3	Kidney	2.51
4	Cancerous liver	4.16
5	Tumor in the hind leg	7.13
